# Functional Groups Dominate Aboveground Net Primary Production under Long-Term Nutrient Additions in a Tibetan Alpine Meadow

**DOI:** 10.3390/plants13030344

**Published:** 2024-01-23

**Authors:** Xueying Chen, Ge Hou, Peili Shi, Ning Zong, Jialuo Yu

**Affiliations:** 1 Key Laboratory of Ecosystem Network Observation and Modelling, Institute of Geographic Sciences and Natural Resources Research, Chinese Academy of Sciences, Beijing 100101, China; chenxueying0845@igsnrr.ac.cn (X.C.); houge0415@163.com (G.H.); zongning@igsnrr.ac.cn (N.Z.); 15527357482@163.com (J.Y.); 2 College of Resources and Environment, University of Chinese Academy of Sciences, Beijing 100190, China

**Keywords:** nutrient additions, functional groups, species diversity, functional diversity, ANPP, Northern Tibetan alpine meadow

## Abstract

Anthropogenic nutrient additions are influencing the structure and function of alpine grassland ecosystems. However, the underlying mechanisms of the direct and indirect effects of nutrient additions on aboveground net primary productivity (ANPP) are not well understood. In this study, we conducted an eight-year field experiment to explore the ecological consequences of nitrogen (N) and/or phosphorous (P) additions on the northern Tibetan Plateau. ANPP, species diversity, functional diversity, and functional groups were used to assess species’ responses to increasing nutrients. Our results showed that nutrient additions significantly increased ANPP due to the release in nutrient limitations. Although N addition had a significant effect on species richness and functional richness, and P and N + P additions altered functional diversity, it was functional groups rather than biodiversity that drove changes in ANPP in the indirect pathways. We identified the important roles of N and P additions in begetting the dominance of grasses and forbs, respectively. The study highlights that the shift of functional groups should be taken into consideration to better predict the structure, function, and biodiversity–ANPP relationship in grasslands, particularly under future multifaceted global change.

## 1. Introduction

During the past few decades, terrestrial ecosystems worldwide have experienced unprecedentedly increasing reactive nitrogen (N) deposition, which originated mainly from fossil fuel burning and artificial fertilizer application, and future global N deposition is expected to increase by 2.5 times or more by the end of this century [1,2]. The increased N deposition has altered plant community composition and ecosystem functioning [3] and also had significant impacts on the biogeochemical cycle [4]. In addition to N, phosphorus (P), as another common limiting element in terrestrial ecosystems, plays an irreplaceable role in affecting ecosystem diversity and productivity [5]. As an important component of terrestrial ecosystems, grasslands account for about 40% of the global land surface. Nutrient additions induced by anthropogenic activities are profoundly affecting the structure and function of grasslands and threatening ecosystem health. However, the underlying mechanisms of these changes remain elusive.

Aboveground net primary productivity (ANPP), as one of the most important ecosystem functions, is largely determined by biodiversity and plant community structure [6]. The biodiversity and functions of grasslands can be affected by both exogenous N and P inputs. Generally, in most grassland ecosystems, N and P additions directly enhance ANPP by supporting plant growth and reproduction due to the decrease in nutrient limitation [5,7,8]. Additionally, nutrient additions alter biodiversity and indirectly affect grassland aboveground biomass [9]. Numerous studies have demonstrated reduction in species richness under N addition due to the imbalance of N:P causing resource competition and soil acidification [10,11]. Also, some have reported that P addition and N + P addition [12] either decrease [13] or have few impacts [14] on species diversity. In addition, previous studies indicated that there was no significant relationship between ANPP and species diversity [15]. It has been argued that species diversity is becoming increasingly less useful in characterizing community structure and serving as a bridge between nutrient additions and ANPP [16]. Over the past decade, a growing interest in functional diversity has spurred a wide investigation into the effects of biodiversity change on ecosystem function [17]. Species within a community have different physiological, morphological, and functional traits; therefore, they contribute in different ways to ecosystem functions. However, species diversity, an indicator that treats all species as equal contributors, is defined without considering the differences in functional traits between individual species. In contrast, functional diversity makes up for the deficiency of species diversity by accounting for the unique functional traits of each species. Thus, this necessitates the incorporation of functional diversity when investigating changes in community structure and ecosystem functions as a result of N and P additions [18]. Achieving higher functional richness with adequately multifunctional or ecological strategies can ensure interspecific niche complementarity and thereby enhance overall resource utilization efficiency and ANPP [19]. However, the effects of N and P additions on functional diversity are unpredictable. Nutrient additions may decrease or increase functional diversity due to a convergence of traits among species [20] or flexible traits in harsh environments allowing more species to coexist, respectively [21]. Moreover, rapid biodiversity loss due to N or P enrichment has attracted interest in investigating how traits variations alter the biodiversity–ANPP relationship [22]. Therefore, further exploration is required to address the effects of nutrient additions on species diversity, functional diversity, and consequently on the changes in ANPP. It seems contradictory that nutrient additions increase ANPP but decrease diversity. Nevertheless, understanding how and why biodiversity and the biodiversity–ANPP relationship change in response to N and P additions is crucial to unveiling the mechanisms by which resources indirectly affect grassland ANPP.

In addition, shifts in functional groups and their compositions also largely influence a community’s productivity. Variations in plant functional groups are commonly observed in response to nutrient additions [23]. The hierarchical response framework (HRF) [24] predicts that chronic resource alterations will have differential effects on functional groups, depending on whether and how a plant community’s composition changes in response to increasing resource availability [25]. In general, exogenous N addition favors grasses rather than legumes in plant communities. On the other hand, P addition inconsistently alters plant functional groups, with either no change [26] or a significant increase in the abundance of forbs [12,27]. The shift in functional groups in response to nutrient additions may be one of the important causes of ANPP alterations, as well as modulating the biodiversity–ANPP relationship.

As a representative of alpine regions around the world, alpine grasslands dominate the Qinghai–Tibetan Plateau, accounting for 1.5 × 10^6^ km^2^, ca. 67% of the total land area there [28]. Alpine meadows are the dominant vegetation type in alpine grasslands [29]. Due to their high altitude, alpine ecosystems are fragile and sensitive to global changes [30]. In recent decades, the alpine grasslands in the Tibetan Plateau have been experiencing an enrichment of N and P [31]. The imbalanced anthropogenic N and P inputs have induced changes in diversity and the plant community structure; however, their ecological effects on productivity are largely unknown. To this end, an eight-year in situ N and/or P addition experiment was conducted in a northern Tibetan meadow to investigate the effects of N and P additions on ANPP, species diversity, functional diversity, functional groups, and ANPP. We aim to explore both the direct and indirect effects of nutrient additions on ANPP. We hypothesized that (1) the nutrient additions would have direct effects on increasing ANPP and that (2) the nutrient additions would lead to changes in species diversity, functional diversity, and the relative abundance of different functional groups, which would modulate the change in the ANPP.

## 2. Results

### 2.1. Effects of N and P Additions on ANPP

**Figure 1:**
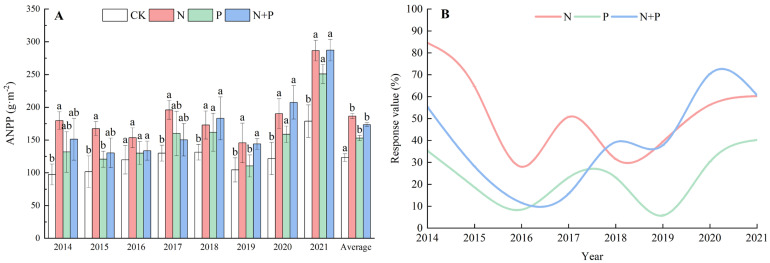
Effects of N and P additions on ANPP (**A**) and response values (**B**). CK, control; N, N addition; P, P addition; N + P, N plus P addition. Different lowercase letters represent significant differences among different treatments in the same year at the 0.05 level.

On average, the N, P, and N + P additions all significantly stimulated the ANPP compared with CK, with an average increase of 51.39%, 24.22%, and 40.80%, respectively (Figure 1A), but the response dynamics varied among the different treatments. It can be seen that the N addition significantly increased ANPP over the whole period, but for the P and N + P additions, significant differences were not shown until the fifth year in 2018 (Figure 1A). The curves of the response values of the N and P additions were approximately similar, tending to slow down from the third year in 2016, while the response value of the N + P addition began to go upwards in the fourth year in 2017 (Figure 1B). Significant effects of the N and P additions on ANPP were observed (Table 1).

### 2.2. Effect of N and P Additions on Species Diversity

**Figure 2:**
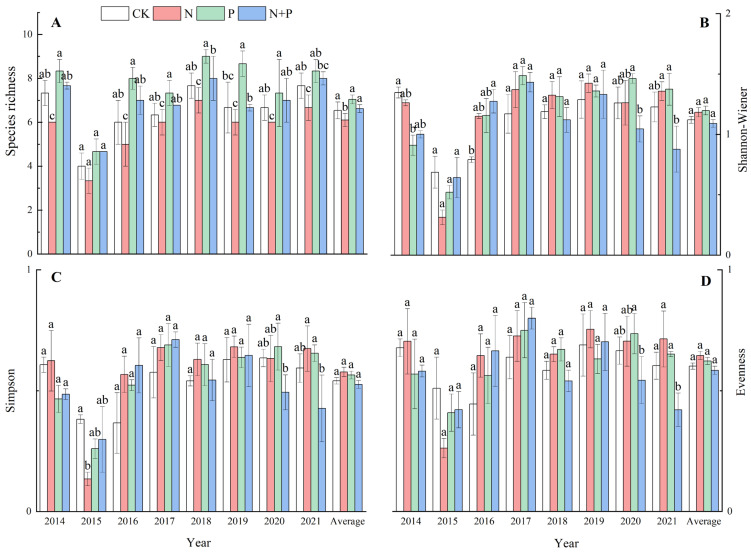
Effects of N and P additions on species richness (**A**), Shannon–Wiener index (**B**), Simpson index (**C**), and evenness (**D**). The abbreviations of treatments and significant differences among different treatments are the same as in Figure 1.

The N addition significantly decreased the species richness (*p* < 0.05, Figure 2A) but had no significant effect on the Shannon–Wiener index or the Simpson index (*p* > 0.05, Figure 2B,C), while it marginally increased evenness (*p* = 0.051) across the eight experimental years (Figure 2D). The P and N + P additions did not have a significant impact on the species richness, the Shannon–Wiener index, the Simpson index, or evenness (*p* > 0.05, Figure 2A–D). Significant effects of the N and P additions on the species diversity were observed (Table 1).

### 2.3. Effect of N and P Additions on Functional Diversity

**Figure 3:**
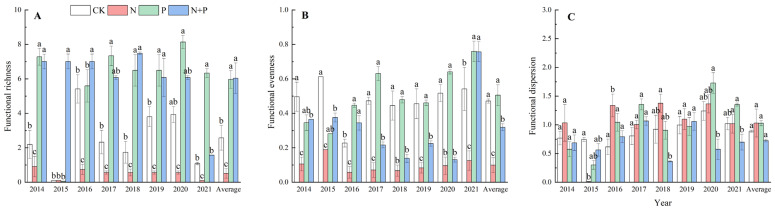
Effects of N and P addition on functional richness (**A**), functional evenness (**B**), and functional dispersion (**C**). The abbreviations of treatments and significant differences among different treatments are the same as in Figure 1.

On average, the N addition significantly decreased the functional richness (*p* < 0.05, Figure 3A) and functional evenness (*p* < 0.001, Figure 3B), but did not change the functional dispersion (Figure 3C). The P addition significantly increased the functional richness (*p* < 0.01, Figure 3A) but had no effect on the functional evenness or functional dispersion (Figure 3B,C). The N + P addition significantly increased the functional richness (*p* < 0.05, Figure 3A), while it decreased the functional evenness (*p* < 0.001, Figure 3B) and had no effect on the functional dispersion (*p* > 0.05, Figure 3C). Significant effects of the N, P additions on functional diversity were observed (Table 2).

### 2.4. Effect of N and P Additions on Functional Groups

**Figure 4:**
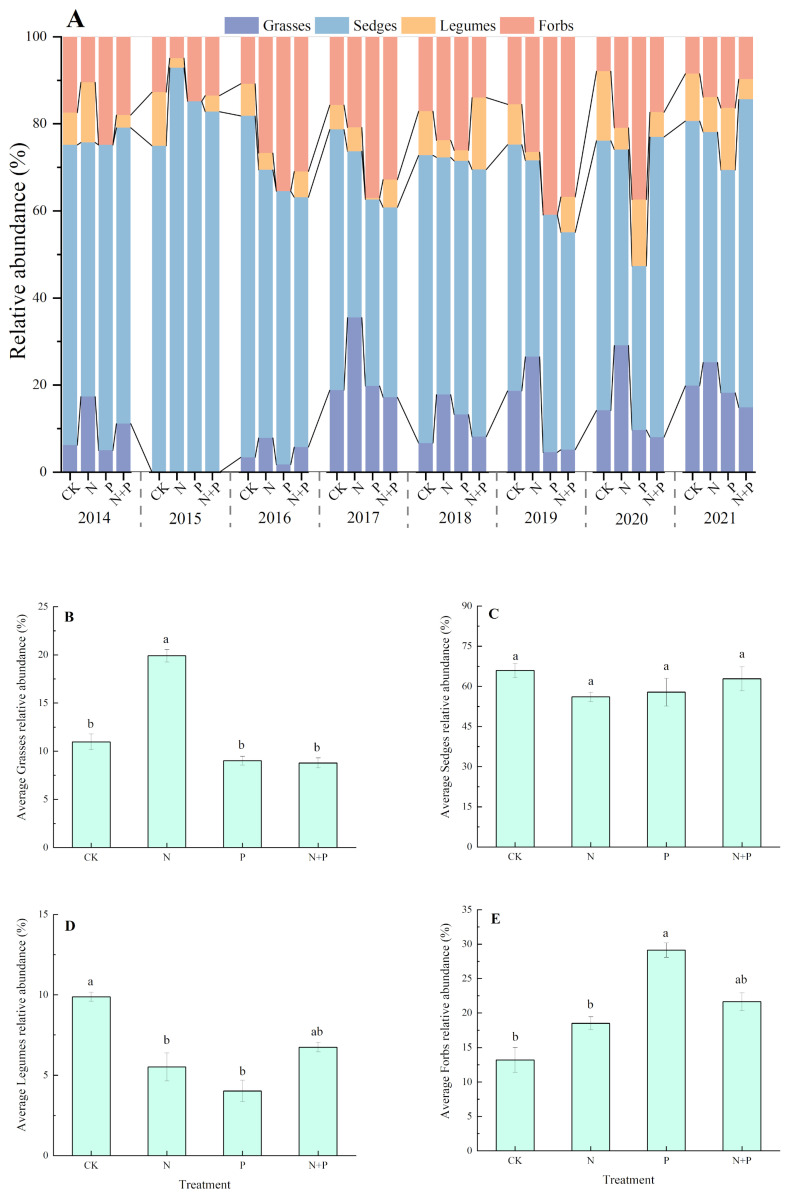
Effects of N and P additions on the relative abundances of four functional groups (**A**) and on the relative abundances, on average, of grasses (**B**), sedges (**C**), legumes (**D**), and forbs (**E**). The abbreviations of treatments and significant differences among different treatments are the same as in Figure 1.

The composition of the plant functional groups was dominated by sedges and grasses, with a low proportion of legumes and forbs in the studied alpine meadow (Figure 4A). The N addition significantly increased the relative abundance of grasses but decreased the relative abundance of legumes (*p* < 0.05, Figure 4B,D), whereas it had no significant impact on the sedge and forbs (Figure 4C,E). The P addition shifted the relative abundance from reducing in legumes to increasing in forbs (*p* < 0.05, Figure 4D,E). The N + P addition had no significant effect on the relative abundance of any of the four functional groups (*p* > 0.05, Figure 4B–E).

### 2.5. Direct and Indirect Pathways of N and P Additions Affecting ANPP

**Figure 5:**
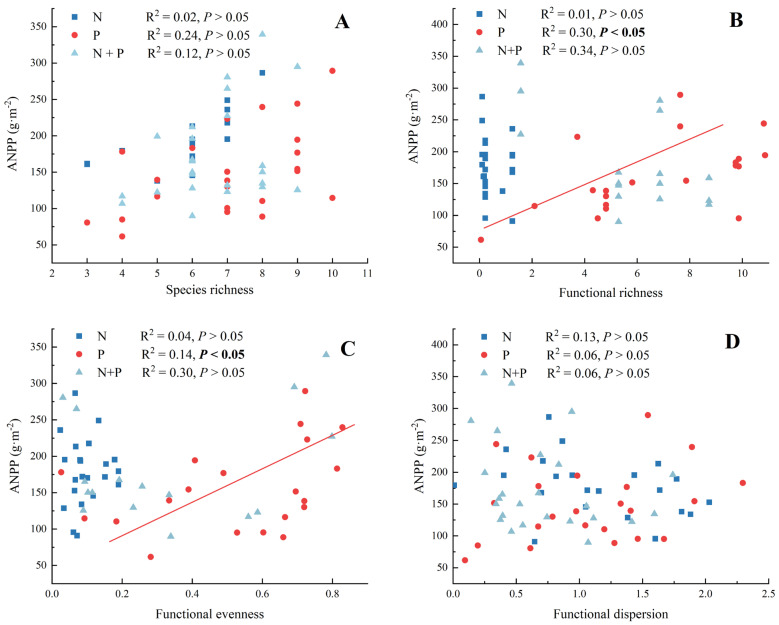
The fitting analysis between species richness (**A**), functional richness (**B**), functional evenness (**C**), functional dispersion (**D**), and ANPP for N, P, and N + P additions. The abbreviations of treatments and significant differences among different treatments are the same as in Figure 1. Significance are bolded.

There were no significant correlations between species richness and ANPP for the N, P, and N + P additions (Figure 5A, *p* > 0.05). In the functional diversity–ANPP relationship, there were significant correlations between functional richness and ANPP (Figure 5B) and between functional evenness and ANPP (Figure 5C) only for the P addition treatment. In addition, there were no effects of any of the components of functional diversity on ANPP for the N + P addition treatment (Figure 5B–D).

**Figure 6:**
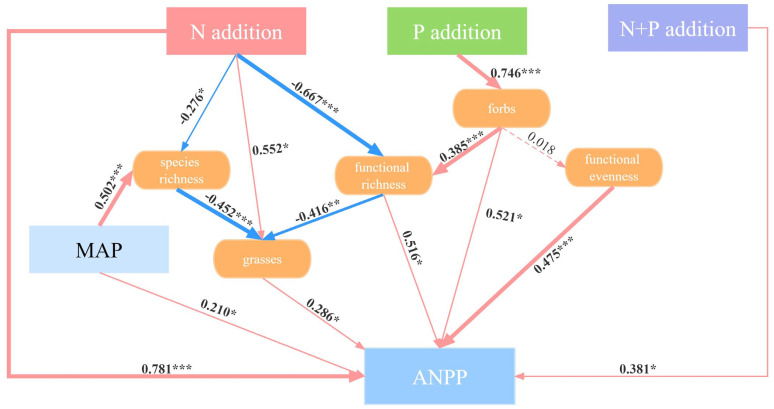
Structural equation modelling to indicate the pathways of the effects of the N, P, and N + P additions on ANPP. Red and blue arrows represent positive and negative pathways, respectively, and solid and dashed lines show significant and non-significant pathways, respectively. The thickness of the arrows indicates the strength of the causal relationship. The final SEM adequately fitted the data: χ^2^ = 6.164; *df* = 3; *p* = 0.104. Significance level: * *p* < 0.05; ** *p* < 0.01; *** *p* < 0.001.

Structural equation modeling (SEM) showed that the N and P additions had different effects on ANPP (Figure 6). The N addition had a significantly direct effect on the ANPP (*p* < 0.001). Also, the N addition promoted the growth of grasses (*p* < 0.05), which in turn increased the ANPP (*p* < 0.05). The decline in species richness (*p* < 0.05) and functional richness (*p* < 0.001) induced by the N addition increased the relative growth of grasses, thereby inducing an increase in the ANPP (*p* < 0.05). In the indirect pathways of the effect of the P addition on ANPP, firstly, the increased relative abundance of forbs (*p* < 0.001) increased ANPP significantly (*p* < 0.05). Secondly, the increase in forbs resulted in an increase in functional richness (*p* < 0.001) and thus increased ANPP (*p* < 0.05). Although forbs did not significantly affect functional evenness (*p* > 0.05), there was a significant positive correlation between functional evenness and ANPP (*p* < 0.001). The N + P addition directly increased the ANPP (*p* < 0.05). Due to inter-annual variation in ANPP, we included the MAP in our analyses, which showed that the MAP was a significant contributor to species richness (*p* < 0.001) and ANPP (*p* < 0.05).

## 3. Discussion

### 3.1. Effect of N and P Additions on the Relative Abundance of Functional Groups

In this study, the N addition significantly increased the aboveground biomass of the plant communities in the alpine meadow (AM), and this increase was mainly caused by the increase in the relative abundance of grasses in contrast to a decrease in the relative abundance of legumes (Figure 4B,D), which is in line with previous results [32,33]. Compared to other functional groups, grasses have greater capacities for nutrient acquisition through the advanced physiological and morphological properties of their roots [34] and the high arbuscular mycorrhizal colonization of their roots in alpine regions [35]. In AMs, grasses are taller in height and have dense root systems, and thus they have an advantage in the competition for water and nutrients [36]. N addition relieves the N limitation of grasses, resulting in their dramatic growth and ANPP enhancement. Exogenous N inputs increase soil N availability, which can potentially reduce the rate of biological N fixation by legumes, consequently decreasing the relative abundance of legumes [37,38].

The P addition had divergent effects on different functional groups in the alpine meadow, represented by a decrease in the relative biomass of legumes and an increase in forbs. Forbs are in the lower part of the plant community, mostly in rosette, semi-rosette, or mat form. P addition can alleviate the limitation of mineral nutrients on the growth of forbs. Also, the vegetation in the study area is sparse and low, and P addition did not significantly increase the relative abundance of grasses, so there was no increase in shade for forbs, resulting in an increase in the relative biomass of forbs. P is also a key regulator of legume growth and metabolism [39]. P limitation affects the construction of legume rhizoma cells and influences energy supply during biological N fixation [40,41]. Because of the high phosphorus consumption during rhizoma development and biological nitrogen fixation, legumes are particularly sensitive to P addition [41]. However, our previous study showed that the studied alpine meadow was not limited by P [42], especially for legumes. In this condition, legumes may not require symbiotic fungi to acquire nutrients, thus reducing carbon allocation to fungi [43]. In addition, an increased abundance of forbs may take advantage of competition over rare legumes and therefore lead to a decrease in the relative abundance of legumes.

In the N + P addition, no significant differences occurred in any of the four functional groups. This may be due to the promoting effect of N and P addition being counterbalanced by the negative effect of shading, for example, resulting in no significant change in the functional groups. Also, we found no significant change in the relative abundance of sedge under any of the treatments, which was the same as other studies in the same region, suggesting that sedges are more adaptable to fluctuations in nutrient resources. 

### 3.2. Effect of N and P Additions on Species Diversity and Functional Diversity

Our findings demonstrated that the N addition decreased the species richness in this AM (Figure 2A), which is in agreement with results obtained in semi-arid steppe [23,44], mountain grassland [45], and alpine grassland [46,47]. The reason for this is because N inputs are mainly related to resource competition and soil acidification [14,48,49]. Generally, N addition relieves plant N limitation but exacerbates light, P, and micronutrient limitations [48]. According to the exclusion theory of resource competition, the convergence of limiting resources among species caused by N addition reduces ecological niche dimensions, intensifies interspecific competition, and ultimately leads to a decrease in community richness [48,50]. An increasing number of studies have shown that fast-growing species, such as the grasses in our study, will dominate after N addition, while the abundance of slow-growing species tends to decline [51]. Moreover, soil acidification caused by N addition is also an important cause of reduced species richness. A decrease in pH due to N addition was observed in this study (Table S1). N addition reduces cation ion exchange, leading to soil acidification and resulting in a decrease in species richness [52,53]. In this study, N addition had a marginally significant effect on evenness (Figure 2D, Table 1), suggesting that the decline in dominant species may be greater than that of subordinate species, indirectly leading to an increase in species evenness. Furthermore, the alterations in species evenness indicated that the N addition changed the community composition [12]. The P and N + P additions did not have a significant effect on species diversity, which is inconsistent with the effect of N addition, suggesting that N addition generally would be a greater threat to diversity loss than P addition in this AM [8]. The impact of P addition on the plant community’s composition was demonstrated by the alteration in the below-ground nutrient competition [54]. P addition could alleviate the negative effect of N deposition on plant species richness in the Tibet AM ecosystem. This result contradicts previous results in European semi-natural grasslands showing that P addition can present a greater threat to biodiversity than N addition [13]. We speculate that soil nutrient availability varies among study sites and has differing effects on biodiversity.

Functional diversity, reflecting the degree of variability in functional traits among species, is a key indicator of biodiversity that has received much attention in recent years. In this study, the N addition significantly decreased functional richness and functional evenness (Figure 3A,B), suggesting that the similarities in functional traits among the species within the community were significant after years of N addition [55]. The intensified competition for resources and the P limitation caused by N addition may contribute to the increase in species competition [48], the reduction in species richness, and the convergence of trait values, leading to a decrease in functional richness. This is consistent with the results of a controlled experiment with multiple levels of N addition in an alpine grassland [56]. In contrast, functional richness was significantly increased under the P and N + P additions (Figure 3A). The increase in functional richness due to the P and N + P additions indicated that the positive effect of plants occupying more functional space in the community was stronger than the negative effect of increasing competition [57,58,59]. Functional diversity is closely related to species ecological niche occupation. The increase in functional diversity after the P addition indicated that the community was more ecologically differentiated and more efficient in its use of resources [60]. On the other hand, after the N + P addition, the functional evenness significantly decreased across the 8 years (*p* < 0.001) (Figure 3B, Table 2), suggesting that the species’ functional traits were closely distributed.

### 3.3. Functional Groups Instead of Diversity Drive ANPP

The N and P additions increased the aboveground biomass, which is consistent with previous studies [5]. We found a highly significant effect of year on the aboveground biomass (*p* < 0.001), which may be related to differences in temperature and precipitation among the years. We calculated the aridity index (AI) to represent the combined effects of precipitation and temperature (Figure S3) and found that there was a high degree of consistency between the AI and ANPP. According to the correlation analysis, the MAP had a significant positive effect on ANPP, while the MAT did not (Figure S4). A fitting analysis of the growing season temperature and ANPP was conducted, and we found that a strong linear correlation only occurred during this period (Figure S5). These results indicated that the MAP was a more significant factor than the MAT in determining the ANPP on the northern Tibetan Plateau. Additionally, we compared the changes in the AI values over the period 2000–2007 and found that the decreasing trend in the AI may be attributed to the rise in the MAT in recent years (Figure S3A). A meta-analysis of 126 N addition experiments across the globe showed that most ecosystems were N-limited, with an average 29% increase in growth in response to N enrichment [7], which was much lower than the average increase in ANPP under the N addition and N + P addition (51.39%, 39.86%) in this study. The results indicated that the studied alpine meadow has a severe N limitation. In contrast to the lack of effect of P addition on increasing ANPP [61], we found that ANPP had increased significantly since 2019, i.e., after 6 years of P addition, indicating that accumulated P enhanced ANPP in the alpine meadow. From the response value (Figure 1B), it can be seen that the values decreased in fluctuation under the N P additions alone, while they increased varyingly under the N + P addition. As the duration of fertilization increases, the response value of ANPP under the N + P addition may exceed that of the N addition alone or the P addition alone. Alpine meadows may be co-limited by N and P in the future. 

We identified a significant role for the relative abundance of grasses in the indirect pathways through which N addition impacted ANPP. Our findings (Figure 4B,D) revealed that the N addition resulted in an 81.5% increase in the relative abundance of grasses, accompanied by a 44.1% decrease in legumes, ultimately leading to an increase in ANPP. We found that although the N addition significantly decreased both species richness and functional richness, they both had no direct impacts on ANPP. Furthermore, a significant correlation was found between the decrease in species richness and the increase in the abundance of grasses, which explained the potentially conflicting phenomena of decreased diversity and increased biomass caused by the N addition. Isbell [10] analyzed the simultaneous dynamics of productivity and diversity as observed in long-term fertilization experiments and revealed that the decline in the trend of increased productivity (direct effect) in grassland communities was associated with the indirect effect of reduced species diversity (non-random species loss). Similarly, a notable contribution of forbs under the P addition was observed. The P addition increased the relative abundance of forbs and also increased functional richness, which in turn increased ANPP. After the N + P addition, the relative abundance of the four functional groups did not change significantly, and although the N + P addition had a significant effect on functional richness and functional evenness, it did not impact ANPP. The increase in ANPP under the N + P addition was due to the release of N and P co-limitation. 

Consistent with the HRF, we focused on the long-term response of different functional groups to N and P additions in our study and indicated that these shifts would drive dramatic changes in ANPP. We did not observe a significantly direct relationship between biodiversity and ANPP in this eight-year manipulation of nutrient additions, similar to the results of a multi-resource addition experiment in temperate grasslands [6]. Perhaps the mediating roles of biodiversity in the effects of N and P additions on productivity need more long-term experiments to be proven [10].

## 4. Materials and Methods

### 4.1. Study Area

This study was conducted on the northern Tibetan Plateau, also called the Changtang Plateau, with an average altitude of ca. 4500 m. The surface of the plateau is characterized by quite integrated, hummocky, uneven terrain, with valleys and basins widely distributed [62]. The alpine meadow (AM) sampling site is located in Nagqu (31°34′ N, 92°34′ E) (Figure S1). The study area is characterized by a typical plateau continental climate, with a mean annual temperature (MAT) below 0 °C, ranging from about −15 °C in the coldest month in January to 10 °C in the warmest month in July. The mean annual precipitation (MAP) is 546.6 mm, mainly concentrated in June through August (Figure S2). The soil type was classified according to FAO as eutric cambisol, with a soil organic matter content of about 4.0%. Herbaceous plants can be divided into four main functional groups, i.e., grasses, sedges, legumes, and forbs. The alpine meadow is dominated by the sedge *Korbresia pygmea* C.B. Clarke and accompanied by the grass *Stipa purpurea* Grised, the legume *Stracheya tibetica*, the forbs *Lancea tibetica* Hook. F. Et Thomas, and other plants [63,64]. 

### 4.2. Experiment Design and Community Survey

The experiment was designed as a randomized block pattern in the most uniformly vegetated area in June 2013. Four treatments were designed as the control (CK), N addition (N), P addition (P), and N plus P addition (N + P), with four replications for each treatment. The 4 × 4 m quadrats were spaced 2 m apart from each other. N and P were added as CO(NH_2_)_2_ and KH_2_PO_4_, respectively, both at a rate of 5 g N m^−2^ yr^−1^. The combined N + P was added at rates of 5 g N m^−2^ yr^−1^ and 5 g P m^−2^ yr^−1^, respectively. Granular fertilizers were applied annually at doses directly before plant green up. All the plots have been fenced off to avoid grazing by stocks and large animals for about 13 years since the implementation of the project “Returning Grazing Land to Grasslands” in Tibet.

A plant community survey was conducted every mid-August from 2014 to 2021, during the peak of the growing season. Specifically, a 1 m × 1 m quadrat in each replicate of the treatments was randomly selected while avoiding that of the previous year’s survey to investigate the number of species, species composition, and community structure. Then, a 0.5 m × 0.5 m sampling area was located in the center of each quadrat to measure the aboveground biomass. The living aboveground parts of each species were cut off from the ground and then packed in paper bags separately. All samples were brought back to the laboratory to determine biomass after oven drying at 65 °C to a constant weight. The biomass in the sampled peak season was considered the ANPP in this studied alpine meadow.

After the aboveground plant survey, soil samples were collected from each quadrat by mixing four drills of soil cores to a depth of 0–15 cm and passing them through a 2 mm sieve to remove roots, litter, and stones. All soil samples were then divided into two subsamples. One was preserved as fresh soil for the determination of available nutrients, and the other was air-dried for the analysis of the pH and total nutrients. 

### 4.3. Soil Analysis

The soil pH was measured in a 2.5:1 mixture of water and soil with a glass electrode meter (InsMarkTMIS126, Shanghai, China). The soil’s total nitrogen (TN) was measured by the Kjeldahl method [65]. Soil NH_4_^+^-N and NO_3_-N were measured using a Seal Auto Analyzer (Seal Analytical, Mequon, WI, USA). The soil’s total phosphorus (TP) was measured using an ultraviolet spectrophotometer [66]. The available phosphorus (AP) was determined by the molybdenum blue method using an ultraviolet spectrophotometer at 700 nm. 

### 4.4. Calculation of Plant Community Metrics

#### 4.4.1. Community Aboveground Biomass, Relative Abundance, and Response Value

The community’s aboveground biomass was indicated by the sum of all species. The species relative abundance was determined by dividing the aboveground biomass of each species by the community’s aboveground biomass [67].

In order to explore the trend of biomass variation annually among treatments, we calculated the response value as follows [68]:

(1) $$Response\;value\left(\%\right)=\frac{X_{t}-X_{c}}{X_{c}}\times 100\%$$

in which $X_{t}$ and $X_{c}$ are the aboveground biomass in the treatment and in the control, respectively.

#### 4.4.2. Species Diversity and Functional Diversity 

Species diversity and functional diversity were calculated using the diversity function of the R 4.0.2 vegan package and FD package, respectively. 

(1) Species diversity

Species diversity was represented by species richness, the Shannon–Wiener index, the Simpson index, and evenness, which were calculated, respectively, as follows [69].

The species richness in this study was counted as the number of species occurring in 0.25 m^2^ sample quadrats.

(2) $$Shannon-Wiener\;index=-\Sigma p_{i}lnp_{i}$$

(3) $$Simpson\;index=1-\Sigma p_{i}^{2}$$

(4) $$Evenness=-\Sigma p_{i}lnp_{i}/lnS$$

Among the variables, *pi* is the ratio of the biomass of species *i* to the total biomass of the community, and *S* is the total number of species in the quadrats.

(2) Functional diversity

Functional diversity was represented by functional richness, functional evenness, and functional dispersion [70].

(5) $$functional\;richness=SF_{ci}/R_{c}$$

Among the variables, *SFci* is the ecological niche occupied by species in the community. *Rc* is the absolute range of eigenvalues.

(6) $$EW=\frac{dist(i,j)}{w_{i}+w_{j}}$$

(7) $$PEW=\frac{EW}{\sum_{i=1}^{S-1}EW}$$

(8) $$functional\;evenness=\frac{\sum_{i=1}^{S-1}\left(PEW,\frac{1}{S-1}\right)-\frac{1}{S-1}}{1-\frac{1}{S-1}}$$

Among the variables, *EW* is the weighted uniformity; *dist*(*i*, *j*) is the species Euclidean distance between species *i* and species *j*; *PEW* is the biased weighted uniformity; and *S* is the number of species.

(9) $$functional\;dispersion=\frac{\sum a_{j}z_{j}}{\sum a_{j}}$$

Among the variables, *a_j_* is the abundance of species *j*, and *z_j_* is the distance from the weighted centroid of species *j*.

#### 4.4.3. Aridity Index

The index was calculated by the following equation [71]:

(10) $$AI=\frac{P}{T+10}$$

where *P* (mm) is the mean annual precipitation (MAP), and *T* (°C) is the mean annual temperature (MAT).

### 4.5. Statistic Analysis

SPSS (SPSS Inc. Chicago, IL, USA) was used for the data analysis. A linear mixed model was used to examine the effects of N, P, N + P, year and their interactions on species diversity, functional diversity, and ANPP, in which the fixed effects were the three treatments (N, P, and N + P) and the year and the random effects were the plots. Also, a one-way ANOVA was used to test the significance of the effects of the N, P, and N + P additions on ANPP, species diversity, functional diversity, and functional group differences in every sampling year based on Duncan’s multiple comparison test (α = 0.05). A structural equation model (SEM) was established using Amos software to determine the direct and indirect effects of nutrient additions on biodiversity and ANPP. The goodness of fit was assessed using χ^2^ and the corresponding *p*-value. ArcGIS 10.2 (Environmental Systems Research Institute, ESRI (Redlands, CA, USA)) was used to make thematic maps (Figure S1). All graphs were drawn using Origin 2023 (Origin Lab Corporation (Northampton, MA, USA)).

## 5. Conclusions

Our eight-year in situ N and/or P addition experiment in an alpine meadow demonstrated how the nutrient additions directly and indirectly affected ANPP. The results showed that the nutrient additions significantly increased ANPP due to the direct release of reduction in nutrient limitation. In the indirect pathways, we found that it was functional groups rather than biodiversity that drove changes in ANPP. The changes in biodiversity did not have direct effects on ANPP, although the N addition had significant effects on species and functional diversity, and the P and N + P additions had significant effects on functional diversity. We found an important role of grasses in affecting ANPP in N addition, as well as a notable contribution of forbs to ANPP under the P addition. Our results provide insights into the nutrient additions–biodiversity–ANPP relationships, which are crucial to advancing our understanding of the multiple facts affecting ANPP in alpine meadows under global changes.

## Figures and Tables

**Table 1:** Linear mixed modelling the effects of N, P, and N + P additions on ANPP, components of species diversity and their interactions with years. Significant differences (*p* < 0.05) are in bold.

Variation	ANPP	Species Richness	Shannon–Wiener	Simpson	Evenness
Year	<0.001 ***	<0.001 ***	<0.001 ***	<0.001 ***	<0.001 ***
N	<0.001 ***	0.022 *	0.240	0.158	0.051
Year × N	0.669	0.557	0.065	0.009 **	0.047 *
P	0.031 *	0.161	0.185	0.417	0.545
Year × P	0.185	0.130	0.029 *	0.155	0.466
N + P	<0.001 ***	0.768	0.633	0.654	0.630
Year × N + P	0.604	0.415	0.019 *	0.046 *	0.093

Significance level: * *p* < 0.05; ** *p* < 0.01; *** *p* < 0.001.

**Table 2:** Linear mixed modelling the effect of N, P, and N + P additions on components of functional diversity and their interactions with years. Significant differences (*p* <0.05) are in bold.

Variation	Functional Richness	Functional Evenness	Functional Dispersion
Year	0.828	0.093	0.048 *
N	0.024 *	<0.001 ***	0.271
Year × N	0.937	0.074	0.229
P	0.007 **	0.642	0.264
Year × P	0.934	0.490	0.403
N + P	0.015 *	0.001 ***	0.142
Year × N + P	0.578	0.013 *	0.334

Significance level: * *p* < 0. 05; ** *p* < 0.01; *** *p* < 0.001.

## Data Availability

Data are available upon a reasonable request.

## Supplementary Materials

The following supporting information can be downloaded at https://www.mdpi.com/article/10.3390/plants13030344/s1: Figure S1: The location of study area; Figure S2: MAP and MAT in the study area from 2014 to 2021; Figure S3: The changes in the aridity index (AI) from 2014 to 2021 (A) and 2000 to 2007 (B); Figure S4: Correlation analysis between MAP, MAT, AI, and ANPP; Figure S5: Fitting analysis of growing season temperature and aboveground biomass; Table S1: Variations in soil properties under four treatments.
