# Supplementary material for: Functional Groups Dominate Aboveground Net Primary Production under Long-Term Nutrient Additions in a Tibetan Alpine Meadow
Source: Plants (Basel). 2024 Jan 23;13(3):344. doi: 10.3390/plants13030344 (PMC10857096; doi:10.3390/plants13030344)
Supplement: Supplementary file 1 [file plants-13-00344-s001.zip › plants-2769592-supplementary.pdf]

Supplementary Information

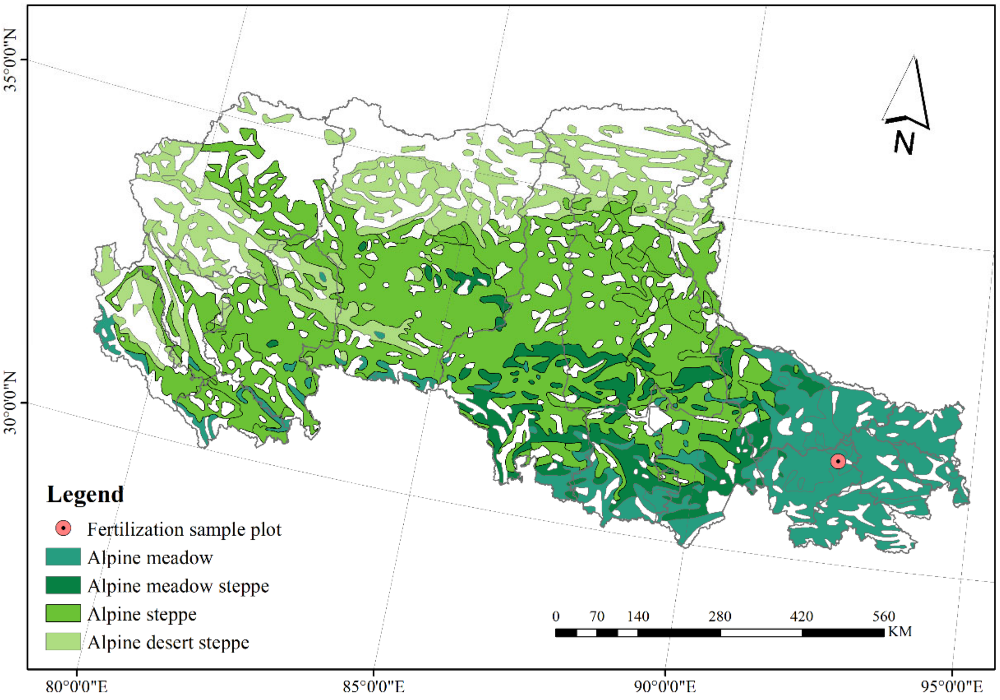

Figure S1. The location of study area

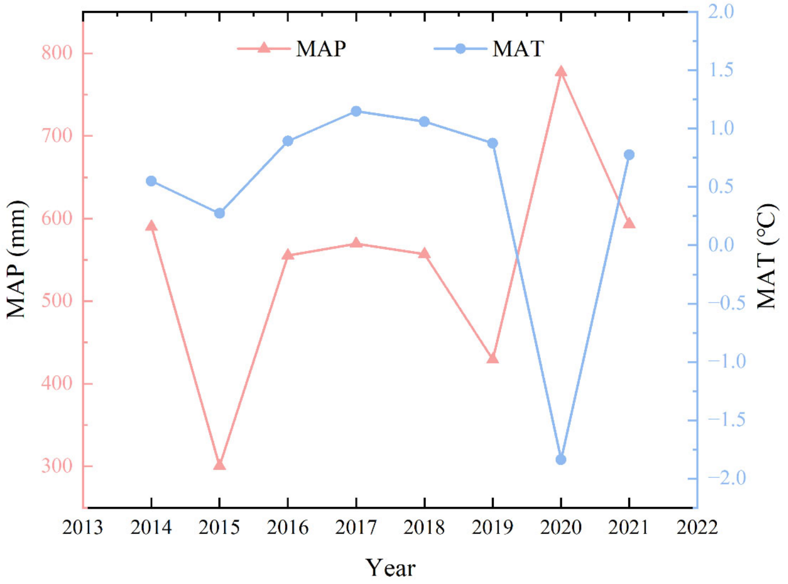

Figure S2. MAP and MAT in the study area from 2014 to 2021

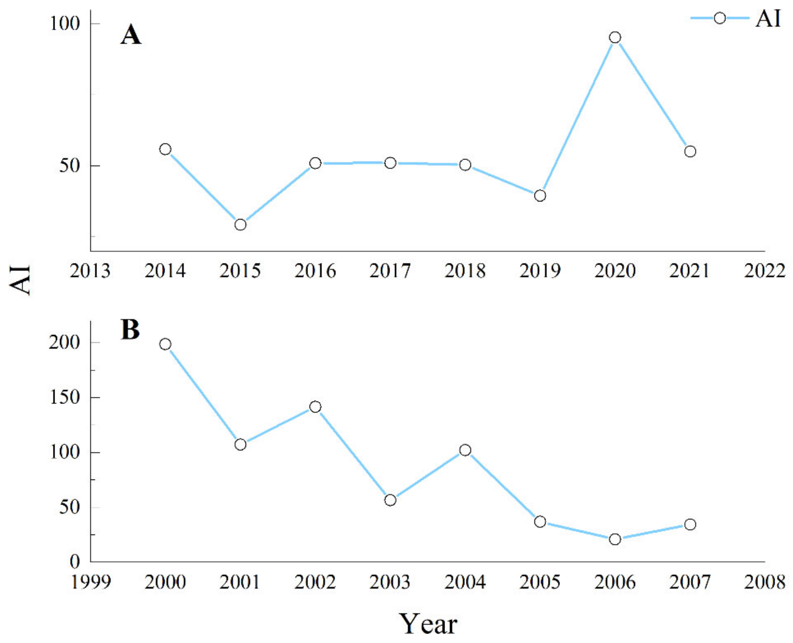

Figure S3. The changes of the aridity index (AI) from 2014 to 2021 (**A**), 2000 to 2007 (**B**).

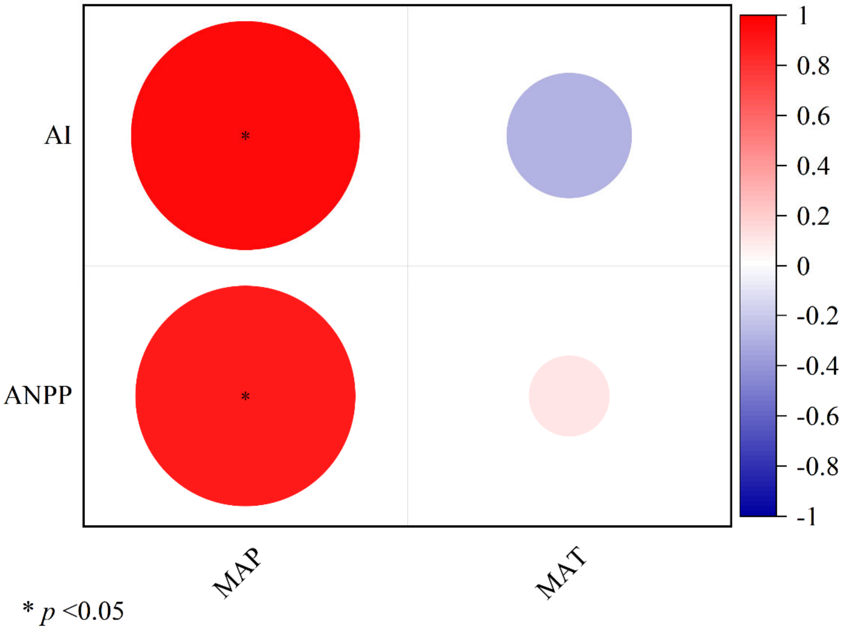

Figure S4. Correlation analysis between MAP, MAT, AI and ANPP.

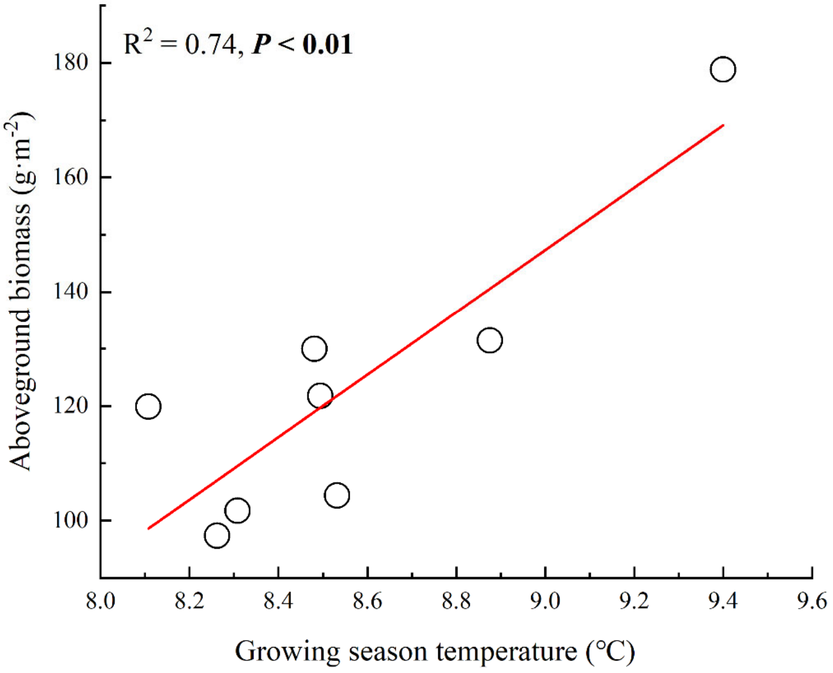

Figure S5. Fitting analysis of growing season temperature and aboveground biomass.

Table S1. Variations of soil properties in four treatments.

**Treatment**	**pH**	**NH_4^+-N (mg·kg^{-1})**	**NO_3^-N (mg·kg^{-1})**	**TP (g·kg^{-1})**	**TN (g·kg^{-1})**	**AP (g·kg^{-1})**
CK	6.7±0.5 a	10.42±4.6 a	15.99±1.4 b	0.32±0.01 b	3.56±0.4 a	8.53±2.9 b
N	6.2±0.5 b	10.43±2.7 a	25.85±3.8 a	0.37±0.05 b	3.91±0.4 a	10.09±2.0 b
P	6.5±0.2 ab	12.23±1.3 a	13.45±3.0 b	0.54±0.04 a	3.90±0.6 a	76.8±16.7 a
N+P	6.1±0.2 ab	12.38±5.9 a	24.34±5.6 a	0.55±0.02 a	3.74±0.4 a	83.80±6.5 a

TN, soil total N; TP, soil total P; AP, available P. Different letters indicate significant differences (Duncan's test, p <0.05) among four treatments.
